# Multifunctional Sponge-like Biochar@ZnO Nanorods Material: Applications in Triboelectric Nanogenerators to Enhance Photocatalysis

**DOI:** 10.1021/acsomega.5c10833

**Published:** 2026-02-16

**Authors:** Agnes Nascimento Simões, Rafael Aparecido Ciola Amoresi, Glauco Meireles Mascarenhas Morandi Lustosa, Waldir Antonio Bizzo, Talita Mazon

**Affiliations:** † Faculdade de Engenharia Mecânica, Departamento de Energia (DE), R. Mendeleyev, 28132 Universidade Estadual de Campinas, 200 - Cidade Universitária, Campinas - SP, 13083-860 Campinas, São Paulo, Brazil; ‡ Centro de Tecnologia da Informação Renato Archer (CTI) Ministério da Ciência, Tecnologia e Inovação (MCTI), Rod. D. Pedro I, KM 143.6, 13069-901 Campinas, São Paulo, Brazil; § Departamento de Química Física y Analítíca, 16748 Universitat Jaume I, Av. Sos Baynat, s/n, 12071 Castellón de la Plana, Spain

## Abstract

Triboelectric nanogenerators (TENGs) have emerged as promising devices for harvesting mechanical energy and enhancing photocatalytic processes, offering innovative pathways toward sustainable technologies. In this study, we report the fabrication of a TENG using environmentally friendly materials. A sponge-like biochar@ZnO nanorods (NRs) composite, synthesized via pyrolysis followed by chemical bath deposition, served as the positive dielectric material, while a PDMS@GO composite formed the negative dielectric layer. The resulting device achieved a power density of 35.11 mW.m^–2^, an output voltage of 7.6 V, a load resistance of 47 MΩ, and a current of 0.16 μA. The TENGs demonstrated excellent reproducibility, delivering consistent voltage outputs across triplicate measurements, and successfully charged a 1000 μF capacitor within 4 h. Beyond energy harvesting, the TENG was integrated into a photocatalytic system to evaluate its performance in degrading methylene blue (MB). When two TENG units were employed, the degradation efficiency reached 42% within 2 h, confirming the synergistic interaction between the TENG’s energy-harvesting mechanism and photocatalysis. These findings demonstrate not only the feasibility of employing sustainable materials in TENG fabrication, in line with circular economy principles, but also the potential of TENG-assisted photocatalysis as a multifunctional strategy for both renewable energy conversion and environmental remediation.

## Graphical abstract

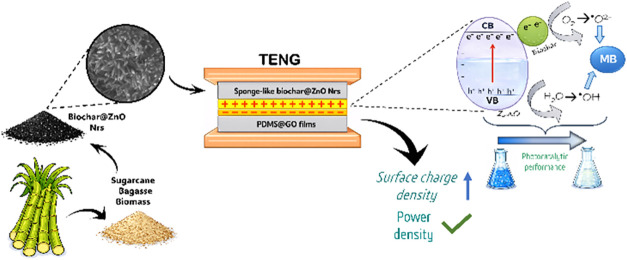

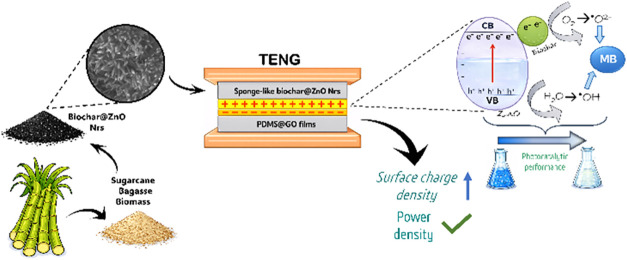

## 1 Introduction

The depletion of fossil fuel reserves, the increasing global demand for energy, and environmental degradation, driven mainly by technological advancements and the miniaturization of electronic devices, necessitate the development of efficient, small-scale power supply systems and self-powered devices. The development of nanogenerators (NGs) offers technological inspiration for improving devices in health, energy, and the environment.^1,2^ NGs mainly include triboelectric nanogenerators (TENGs), piezoelectric nanogenerators (PENGs), and thermoelectric nanogenerators. These devices convert energy from mechanical, thermal, and chemical processes into electrical energy.^3^ By converting mechanical energy (e.g., body movement, wind, and water waves) into electricity, TENGs and PENGs offer promising solutions for powering conventional portable electronics. These devices mainly differ in their final structure and fundamental working principle for generating electricity: PENG consists of a single piezoelectric material that, when bent, stretched, or compressed, produces an electrical potential within it; in contrast, the TENG’s electrical property results from contact electrification and separation of two different materials, creating a potential difference between them and causing a transfer of charge carriers.^4,5^ The TENG static polarization of charges on the electrode surfaces enables the conversion of mechanical energy into electrical energy.^6,7^ In addition to their interesting performance, simple fabrication, lightweight structure, and the possibility of using low-cost, environmentally friendly materials, TENGs are efficient, sustainable devices for converting low-frequency motions into electricity.^6–8^

Various materials exhibiting distinct charge affinities can be used to construct electrodes for the TENG devices. These materials are classified by polarity in the triboelectric series and offer numerous opportunities for further research, owing to their natural ability to donate (positive dielectric) or accept (negative dielectric) electrons.^9^ They include: metals,^10^ polymers,^11^ 2D materials,^12^ crystalline oxides,^13^ and carbon materials.^14^ Carbon-based nanomaterials, such as graphene and graphene oxide (GO), are promising candidates and have attracted considerable attention in research and the development of recent technologies. GO is a form of graphene containing oxygen with an electronic bandgap suitable for photonic applications.^15^ The electronic structure of GO, particularly the relationship between the π electrons of benzene rings and the degree of oxidation in GO layers, influences the energy gap between the highest occupied molecular orbital (HOMO) and the lowest unoccupied molecular orbital (LUMO).^16^ This relationship suggests that controlling the degree of oxidation or GO’s interaction with π-electron donor/acceptor materials^17^ can regulate the charge-transfer process, making GO ideal for photonic or photocatalytic applications.^16,18^ Another environmentally friendly carbon-based material receiving attention is biochar (BC), obtained by pyrolyzing biomass, an abundant and renewable raw material. Biochar has a high carbon content, a stable structure, alkalinity, abundant oxygen-containing functional groups, a high cation exchange capacity, and a porous structure with a high surface area, which enable its use in environmental sciences.^19,20^ Its large surface area enables (i) increased charge-adsorption capacity and, consequently, improved energy-generation efficiency in TENGs,^21,22^ (ii) possibility of incorporating metals or metal oxides on its surface results in removing contaminants from wastewater by adsorption or photodegradation,^23,24^ and (iii) oxy-reduction behavior,^25,26^ making it promising in catalysis. Notably, biochar is more cost-effective than many other carbon nanostructures, as it can be easily produced from natural sources (organic, agricultural, or sewage) through physical or chemical methods.^27^

The incorporation of metal oxide semiconductors (MOS) into biochar matrices has proven to be a promising strategy for optimizing electrical properties.^28,29^ MOS exhibits tunable electrical conductivity and, when anchored in the biochar matrix, forms a network that facilitates charge transport, increasing the efficiency of converting mechanical energy to electrical energy. Additionally, this approach can induce stronger interfacial polarization, increasing the surface charge density and, consequently, the TENG’s power output and confer greater chemical and mechanical stability to the biochar, thereby expanding the device’s durability and resistance to adverse environmental conditions. ZnO is a well-known piezoelectric material; i.e., it generates an electric potential in response to mechanical stress. The main contribution of zinc oxide in a TENG device is to increase the composite’s dielectric constant, which allows more charge accumulation on the TENG’s surface during the contact and separation process, boosting the overall permittivity of the matrix.^30,31^

The power generated by the TENG can be directly used for wastewater purification, self-cleaning systems, and self-sufficient electrocatalytic technology. Therefore, it enables the effective removal of pollutants from wastewater without any additional consumption.^32,33^ In this way, photocatalysis is a sustainable method for degrading organic pollutants and can be activated by harnessing solar or mechanical energy.^34,35^ Some studies explore the use of TENGs for photocatalysis as emerging, promising alternatives for self-powered degradation of chemical pollutants.^36^ Su et al.^37^ combined TiO_2_ nanoparticles in a TENG device attached with a platinum (Pt) electrode, PTFE (polytetrafluoroethylene) film, and aluminum (Al) foil as the friction layers. They observed that after two h, the degradation achieved was 76% of the Methyl Orange dye, higher than 26% obtained without the TENG. Chen et al.^38^ report results in removing almost 100% of Rhodamine B dye in 15 min from an initial concentration of 100 ppm. They comprised a graphite anode, an iron cathode, and 20% (w/v) NaCl as the electrolyte to enhance ionic strength; however, some byproducts can be generated, and intermediate organic products, such as carboxylic acids, may also be present. Dong et al.^39^ developed a TENG device with TiO_2_ nanoparticles to improve the generation of hydroxyl radicals (^•^OH) and then promote the catalysis of Atrazine (ATZ) pesticide. The removal rate by the photocatalytic TiO_2_ nanosheets was 41.8%, whereas upon the introduction of pulsed direct current generated by a TENG, the removal rate increased to ∼52%. When a photoelectrode with TiO_2_ nanotubes was introduced into the system, the TENG devices achieved an AZT removal rate of over 90%. Despite advances, TENGs used in photocatalysis have not yet been developed to improve material efficiency in real effluents, to evaluate service life, or to analyze the dependence of materials on irradiation wavelength and light intensity.

Therefore, the development of high-performance TENG devices requires materials optimized for strong triboelectric charge generation, enhanced power density through optimized device design, and environmentally friendly, multifunctional materials. Despite the promising potential of TENGs, several challenges hinder their widespread application. Achieving a low-cost, straightforward fabrication process that enables scalable production remains a key obstacle. Enhancing electrode contact to maximize the triboelectric effect and increasing the power output of these devices remain active areas of research. By converting mechanical energy into electricity, TENGs can enhance the photocatalytic performance. This integration is promising for the development of more efficient and sustainable technologies for environmental remediation. This synergistic combination exploits the properties of both materials, resulting in devices with enhanced performance. Although biochar-supported materials have been extensively studied for their ability to degrade organic pollutants and for use in triboelectric nanogenerators (TENGs) for energy harvesting, few studies have explored the integration of biochar-based photocatalysis with biochar-based triboelectric power to enhance photocatalytic processes.

Here, we propose integrating biochar-based composites with sponge-like microstructure into TENG devices for use as photocatalysts in sustainable environmental remediation. A multifunctional, environmentally friendly, sponge-like biochar@ZnO NRs composite was employed as a key component in the TENG architecture. The sponge-like biochar@ZnO NRs composite served as the positive dielectric, while a polydimethylsiloxane (PDMS) film containing graphene oxide was used as the negative dielectric. The sponge-like microstructure of the biochar@ZnO NRs composite enhances surface area and effective contact, promoting increased triboelectric charge accumulation,^40^ and acts as a charge-transport mediator, increasing the free-carrier concentration.^41,42^ PDMS, a low-toxicity, biocompatible silicone-like material widely used in triboelectric applications,^43–45^ was selected as the negative dielectric due to its high electronegativity, flexibility, and optical transparency.^46–48^ The integration of the sponge-like biochar@ZnO NRs composite into the TENG significantly improved charge transfer and power-generation performance, enabling its effective application in photocatalysis.

## 2 Experimental Procedure

### 2.1 Raw Materials

Phosphoric acid (H_3_PO_4_ – Dinâmica), sulfuric acid (H_2_SO_4_ – Dinâmica), hydrochloric acid (HCL – Dinâmica), zinc acetate (Zn­(CH_3_CO_2_)_2_ (ZnAc) – Sigma-Aldrich), zinc nitrate hexahydrate, (Zn­(NO_3_)_2_·6 H_2_O – Sigma-Aldrich), ammonium hydroxide (NH_4_OH – Dinâmica), polyvinylidene difluoride (PVDF – Sigma-Aldrich), n-methyl pyrrolidone (NMP – Sigma-Aldrich), polydimethylsiloxane Sylgard 184 (PDMS – Dow Corning), methylene blue (Ecibra), sugarcane bagasse biomass (in natura from São Paulo region), and GO (synthesized via modified Hummers’ method in our laboratory, by using methodology previously reported^49^). All commercially available chemicals were of analytical grade.

### 2.2 Materials Processing

#### 2.2.1 Preparation of the Biochar@ZnO NRs

First, sugarcane bagasse biomass was submitted to acid treatment with H_3_PO_4_ and H_2_SO_4_ in an autoclave recipient at 80 °C for 3 h under constant stirring. After cooling, the product was washed with distilled water until the pH was neutral. Then, the biomass was calcined at 750 °C for 4 h under a N_2_ atmosphere at 3 mbar. The carbonized powder (namely, biochar) was then placed in a 0.5 M HCl solution for 1 h under constant stirring at room temperature, followed by washing and filtration steps until the pH was neutral. The resulting powder was dried overnight in an oven at 100 °C.

The biochar was macerated and sieved through 500 and 74 μm sieves. The powder was placed in an 80 mM ZnAc solution in ethanol under constant stirring at room temperature for 1 h to generate active zinc sites on its surface and then put in an oven at 100 °C overnight to promote complete solvent evaporation. Zinc oxide NRs were grown onto the biochar surface through the chemical bath deposition method (CBD) at 90 °C/2 h by using NH_4_OH (6.5 mL) and Zn­(NO_3_)_2_·6 H_2_O (1.724 g) as precursor agents. Finally, the suspension was centrifuged at 10,000 rpm for 30 min and then washed with distilled water. The obtained composite powder was dried overnight at 100 °C and stored for future use.

#### 2.2.2 Preparation of the Sponge-like Biochar@ZnO NRs

The preparation of the sponge-like biochar involved initially dispersing 0.8 g of the biochar@ZnO NRs nanocomposite in approximately 2 mL of NMP together with PVDF. Different PVDF contents were evaluated to ensure the structural stability of the sponge-like biochar, as summarized in Table 1. Initially, PVDF was dissolved in NMP under magnetic stirring at room temperature, followed by the addition of the biochar@ZnO NRs nanocomposite. The mixture was stirred for 5 min to achieve complete homogenization. Then, a polyurethane matrix (PU) (7 × 5 × 5 mm^3^, cut from a commercial sponge) was immersed in the suspension to absorb the composite, and the suspension was left undisturbed overnight at room temperature. The impregnated samples were then subjected to different heat-treatment conditions, as outlined in Table 1, yielding the sponge-like biochar@ZnO NRs structures. Finally, the resulting sponge-like biochar@ZnO NRs was mounted on adhesive copper tape (Cu tape) and used as the positive dielectric material in the TENG. The overall fabrication process of the sponge-like biochar@ZnO NRs-based material is schematically illustrated in Figure 1a.

**Figure 1:**
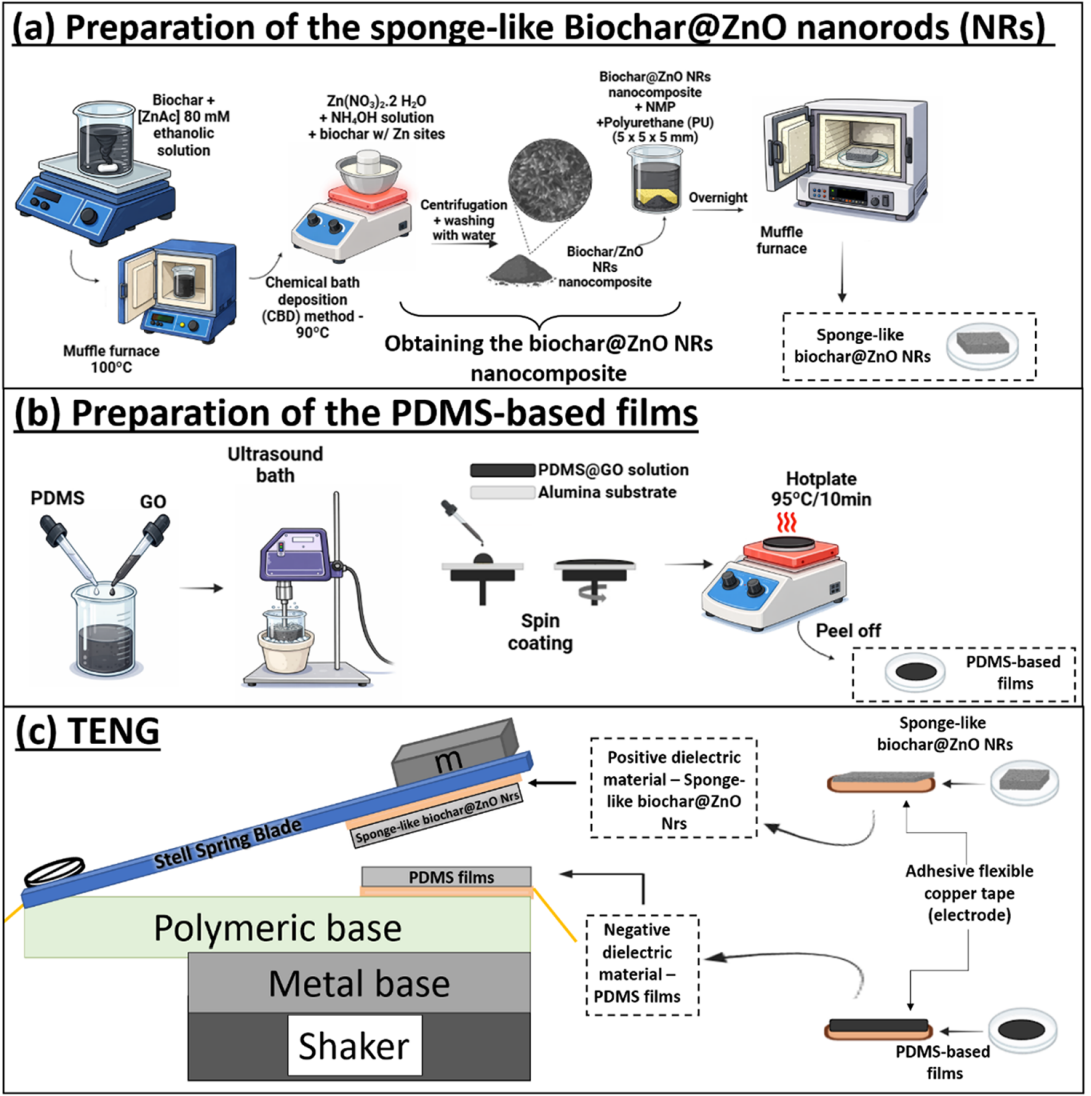
Schematic view of the preparation of (a) biochar@ZnO NRs sponge-like structure, (b) PDMS or PDMS@GO-based films, and (c) assembly of TENG device.

**Table 1:** Conditions Used to Prepare the Sponge-like Biochar@ZnO NRs

sample	PVDF (% w/w)	thermal treatment (°C)	time
E1	5	100	overnight
E2	20	100	overnight
E3^a^	20	200	2 h

a In sample E3, there was a preliminary step at 100 °C/2h.

#### 2.2.3 Preparation of the PDMS-Based Films

Two types of PDMS films, (i) pure and (ii) containing graphene oxide, were prepared to be used as negative dielectric materials in TENG. The PDMS films were prepared from Sylgard 184 at a 1:10 ratio. To obtain the PDMS@GO film, 4 wt % GO sheets were added from a GO suspension at 3.75 mg/mL to the PDMS solution, and the mixture was sonicated to ensure dispersion of the GO sheets. GO sheets suspension was prepared as previously reported.^67^ Then, PDMS and PDMS@GO solutions were deposited onto an alumina (Al_2_O_3_) substrate by spin coating at 3000 rpm for 30 s, and the films were cured at 95 °C on a hot plate. The films were cut into 10 × 10 mm^2^ squares and fixed to an adhesive Cu tape for use as a negative dielectric material in TENG. A flowchart of the steps in obtaining the PDMS-based films is shown in Figure 1b.

#### 2.2.4 . Assembly of TENG Devices

The TENG devices were assembled to operate in a vertical contact-separation mode, as shown in Figure 1c. The Cu tape was used as an electrode (current collector). The biochar@ZnO NRs sponge-like structure acts as the electron-donating material, while the PDMS or PDMS@GO films act as the electron-receiving material. To prepare the device, the PDMS or PDMS@GO films on Cu tape were fixed in a PVC plate (located on the shaker), the sponge-like biochar@ZnO NRs on Cu tape was first soldered onto a steel spring blade (0.5 × 4.7 × 1.3 mm^3^), and then the steel spring containing the material was attached to a polymeric base on the PDMS/Cu/PVC/shaker.

### 2.3 Characterization

#### 2.3.1 Morphological and Structural Characterizations

The crystal structure was characterized by X-ray diffraction (XRD, XRD-7000 Shimadzu) at room temperature using Cu Kα radiation (λ= 1.5460 Å) in the 2θ range from 10° to 80°. The XRD patterns of the samples were identified by using the ICDD database cards. The morphology (size, shape, and porosity) was evaluated by using scanning electron microscopy (SEM) with a Tescan Mira 3 XMU microscope. It was operated at an acceleration voltage of 3 kV for secondary electrons (SE). Fourier-transform infrared spectroscopy (FTIR) and Raman Spectroscopy were used to analyze the chemical composition and structure of the samples. FTIR analyses were performed using a transmittance module in the spectral range of 4000–400 cm^–1^ and a spectral resolution of 4 cm^–1^, on a PerkinElmer Spectrum 100. The Raman measurements were obtained in the 100–4000 cm^–1^ range, with 5 scans, using a Horiba Jobin Yvon Spectrometer model T64000 with a 532 nm (Coherent) laser as the excitation source.

#### 2.3.2 Mechanical TENG Characterization

A “mass-spring” system was used to characterize the TENG system and improve its performance.^68^ As a mass-spring device, it is possible to find the natural or resonance frequency of the system given by eq 1.

1 $$f=\frac{1}{2\pi }.\sqrt{\frac{k}{m}}$$

where *k* is the spring constant and *m* is the spring mass.

In this work, the spring material used was SAE 1070 carbon steel. The frequency was fixed at 60 Hz because this frequency is commonly encountered across environments. Based on the carbon steel SAE 1070 properties and a 60 Hz frequency, we used eq 2 to determine the mass of carbon steel SAE 1070 required to reduce the resonance frequency of the system. We found a spring mass of around 6.5 g.

2 $$f=\frac{1}{2\pi }.\sqrt{\frac{{E.b.e}^{3}}{({4L}^{3})\left(m+0.24m_{b´}\right)}}$$

where *f* is the desirable working frequency (in our case, 60 Hz), *E* is the elasticity coefficient of the spring, *b* is the sprinǵs width, *e* is the sprinǵs thickness, *L* is the sprinǵs length, *m* is the mass to be attached, and *m*_*b*’_ is the spring mass.

#### 2.3.3 TENG Energy Harvesting

Electrical characterization was performed using a vibration generator (“shaker”) (DTC TEN-V20) coupled to an amplifier (DTC TEN-A100). The voltage data generated by the device were collected by using an oscilloscope (Tektronix TDS2014B). Details about the external circuit connected to the triboelectric nanogenerator can be found in our previous work.^68^ The measurements were performed at 60 Hz, with varying resistance load in the external circuit. From the result obtained (voltage) and the device’s active area, it is possible to obtain the power density value (mW/m^2^). To simulate the maximum open-circuit voltage (*V*_oc_) and the maximum short-circuit current (*I*_sc_), a 600 MΩ resistor and a 1 kΩ resistor were used in the external circuit, respectively. A circuit was built with a 1000 μF capacitor and a 1 MW resistor load, and then, the charged voltage was measured by a digital multimeter.

#### 2.3.4 Photocatalytic Property

The photocatalyst activity of the biochar@ZnO NRs was evaluated by photodiscoloration of a methylene blue (MB) aqueous solution (200 mL, *c* = 4 mg/L) under natural ultraviolet light and a 30 W LED flexible ring light placed near the measuring system. Photos of the experimental setup for the photocatalytic activity measurements and TENG operation are shown in Figure S4. The system contains an acrylic recipient housing the built TENG device and the MB aqueous solution. The recipient was sealed with a proper lid, and the TENG device was activated by a mechanical external force (a shaker) at 60 Hz. The parameter “gain” of the amplifier was set to 1.5 V to control the movement amplitude. Solution aliquots (∼5 mL) are collected at predetermined times (0, 15, 20, 45, 60, 90, and 120 min), placed in a quartz cuvette (1 × 1 cm^2^), and characterized by the UV–vis spectrometer Horiba Duetta in the 200–800 nm range to evaluate the MB degradation. The photocatalytic degradation experiments were conducted in two configurations to evaluate the influence of the triboelectric nanogenerator: the first used a single-TENG unit, while the second used two TENG units.

## 3 Results and Discussion

### 3.1 Biomass Characterization

The acid treatment, a crucial step before pyrolysis, is designed to reduce the degree of polymerization and generate a porous surface. Figure S1 illustrates the FTIR spectra, and Figure S2 shows SEM images of sugarcane bagasse biomass before and after the acid treatment and pyrolysis.

In the FTIR spectra, as shown in Figure S1, we can observe for both sample bands at 1730 cm^–1^, 1610 cm^–1^, 1515 cm^–1^, 1020 cm^–1^, and 825 cm^–1^, which are attributed to the C=O stretch of the hemicellulose structure, the aromatic stretch of the lignin molecule, the C=C bond of aromatic compounds also in the lignin molecule, C–O–C vibration characteristic of cellulose and hemicellulose molecules, and the aromatic ring stretch of the lignin, respectively. However, there is a reduction in the intensity of these characteristic bands of structures present in aromatic lignin components of the pure biomass after acid treatment and pyrolysis, indicating the effectiveness of the acid treatment in weakening/degrading the polymeric structure to promote the calcination of the raw material. The most evident observations of the spectral change after acid treatment include the reduction in the bands at 1734 cm^–1^ and the region of 2985–2815 cm^–1^ stands out, the former indicating the efficient cleavage of the acetyl group of the hemicellulose of the lignin components, and the second related to vibrational stretches −C–H (groups −CH_3_, −CH_2_, and −CH) present in lignocellulose fractions.^50^ Such characteristics indicate a decrease in the degree of polymerization of the biomass.

Figure S2 presents SEM analyses of the biomass before and after acid treatment. A fibrous microstructure is observed (Figure S2a) with a morphology of smooth edges and the presence of a few defects, such as half-open pores (Figure S2b). After the acid treatment (Figure S2c,d), the biomass exhibited greater roughness, an irregular surface, and clear pore openings.

### 3.2 Sponge-like Biochar@ZnO NRs Characterization

The microstructure of the sponge-like biochar@ZnO NRs was evaluated using FTIR and SEM. In the IR spectra (Figure 2a), we observed bands at 2975 cm^–1^, attributed to the secondary amide group of the solvent, and at 1700 cm^–1^, characteristic of C=O stretching from the solvent and from the hemicellulose of the biochar. The peaks between 1370 cm^–1^ and 930 cm^–1^ were related to the vibrational modes of the PVDF in its different conformations (α, β, and γ).^51^ In Figure 2b, the spectrum of sample E2 is similar to that of E1 because the heat-treatment temperature is the same for both samples, with the E2 sample showing a higher concentration of PVDF. However, for the E3 sample, upon heating to 200 °C, the most PVDF-related bands were suppressed, indicating polymer melting,^52^ an amorphization of the chains with a prevalence of some α and β crystallites,^53^ as observed in the bands at 1370 cm^–1^ and 1080 cm^–1^.

**Figure 2:**
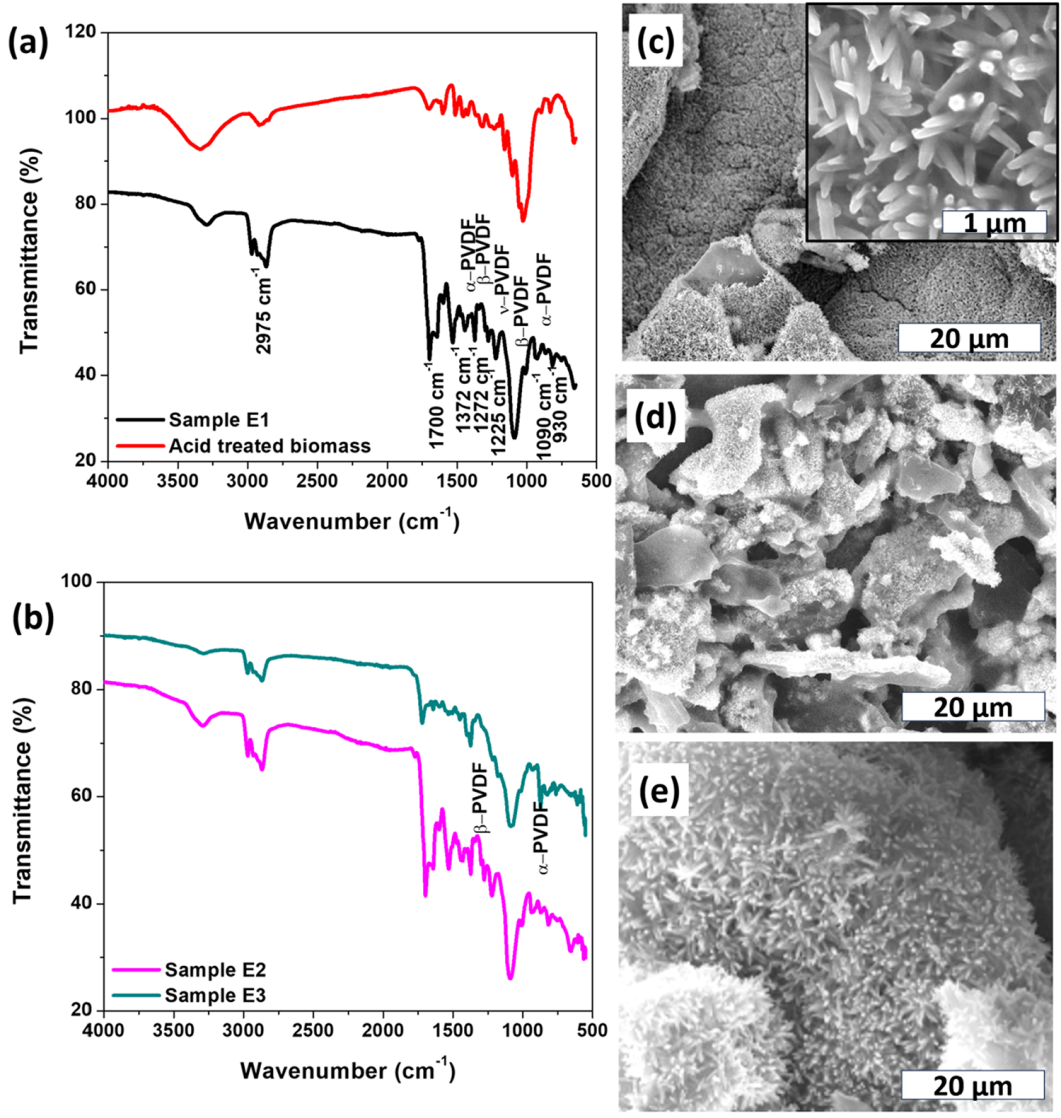
FTIR spectra for biomass and sponge-like biochar@ZnO NRs samples. (a) Biomass obtained after acid treatment and the E1 sample. (b) E2 and E3 samples. SEM images for sponge-like biochar@ZnO NRs samples: (c) E1, (d) E2, and (e) E3.

Figure 2c–2e show the microstructure of the sponge-like biochar@ZnO NRs. In all images, ZnO NRs are observed on the biochar surface, even after the composite was impregnated into the sponge and heated. The ZnO NRs have an average length of 1 μm and a diameter of 5 nm. Furthermore, SEM images show a sponge-like structure with a uniform pore-size distribution for all preparation conditions. However, increasing the PVDF amount and temperature were necessary to produce more stable sponge-like biochar@ZnO NRs.

The as-synthesized biochar@ZnO NRs were structurally analyzed by using XRD and Raman spectroscopy. From the diffraction pattern shown in Figure 3a, the broad peak between 15° and 30° is indexed as a C < 002 > diffraction peak associated with an amorphous carbon structure of the biochar phase.^54^ The well-defined peaks at 31.7°, 34.4°, 36.2°, 47.5°, and 56.6° were attributed to (100), (002), (101), (102), and (110) lattice plans, respectively, of the crystalline zinc oxide of hexagonal phase (ICDD, n° 34477) with space group P63mc. A preferential growth on the (101) plane is observed, consistent with the higher intensity characteristic of uniaxial nanorod growth.^55^ By using the Scherrer equation ( $D=0.9\times \frac{\lambda }{\beta }\cos\theta$ , where λ, β, and θ are the X-ray wavelength, the width at half height of the diffracted peak, and the diffraction angle, respectively), the crystallite size, *D*, was calculated as 4.57 Å, indicating the nano size for the as-synthesized ZnO.

**Figure 3:**
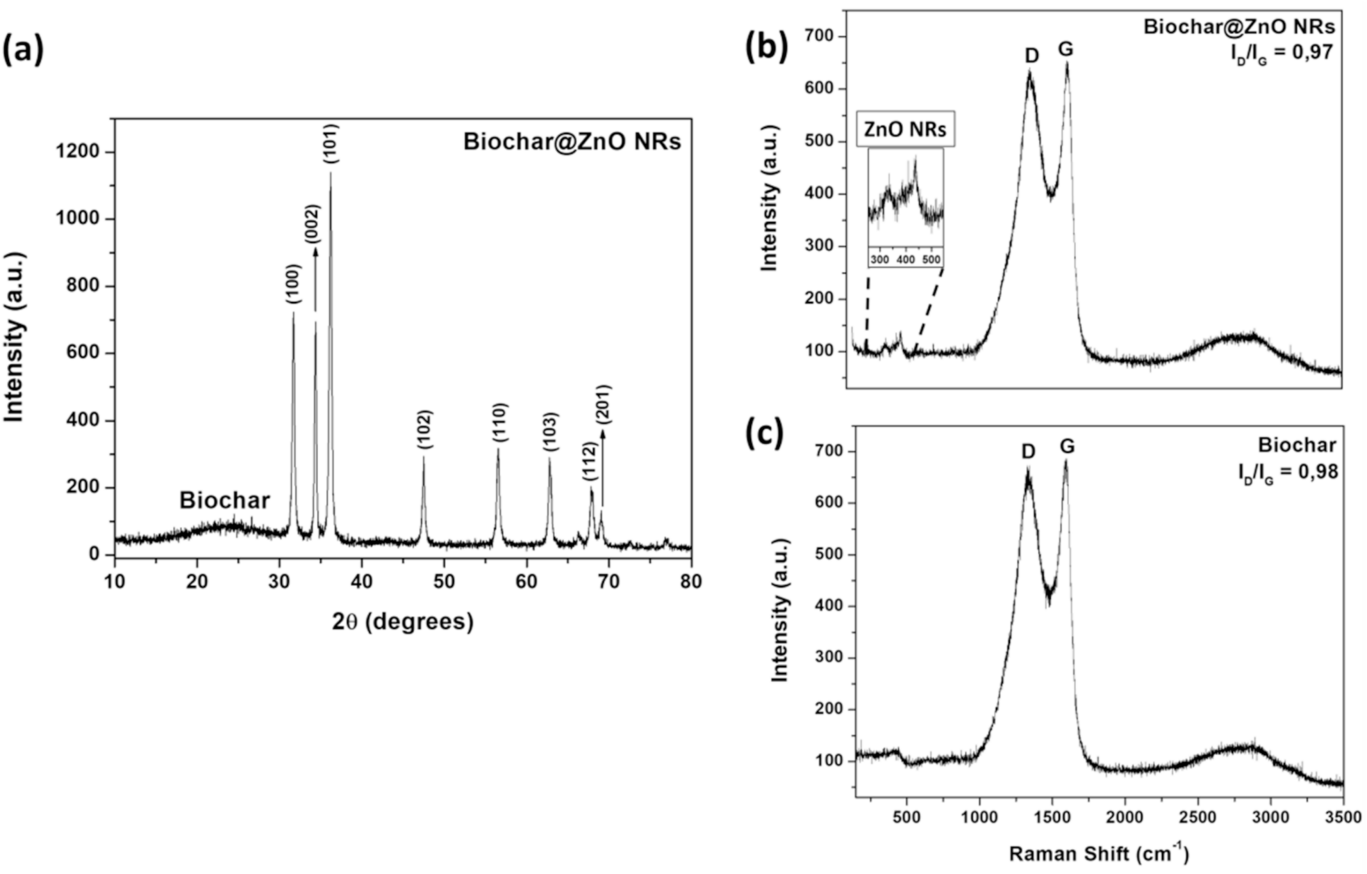
(a) XRD for biochar@ZnO NRs. Raman spectra for (b) biochar@ZnO NRs and (c) pure biochar.

Figure 3b,c show the RAMAN spectra of biochar@ZnO NRs and biochar powders. Two bands between 300 cm^–1^ and 450 cm^–1^ were observed, which refer to the ZnO scattering modes. The band at 332 cm^–1^ is attributed to the second-order mode arising from zone-boundary phonons in hexagonal ZnO, and the band at 438 cm^–1^ is related to the E_2_ symmetry mode, both belonging to the special group P63mc of the hexagonal wurtzite structure of ZnO, corroborating the XRD results.^56,57^ For both samples, we observed bands at 1350–1470 cm^–1^ and 1580–1600 cm^–1^, characteristic of D and G bands, respectively. These bands originate from in-plane vibrations of sp^2^ bonds in carbon structures with structural defects (D band) and in graphitic carbon structures (G band).^58^ The relation *I*_D_/*I*_G_ shown by the biochar and biochar@ZnO NRs nanocomposite (<1) indicates a high level of graphitization.^59^

### 3.3 PDMS-Based Film Characterization

Micrographs of the pure PDMS and PDMS/GO films are presented in Figure 4a and b, respectively. The analysis reveals a significant difference between pure PDMS and PDMS/GO films. Figure 4b demonstrates that the curing process yields well-integrated, evenly distributed GO sheets within the polymer matrix.

**Figure 4:**
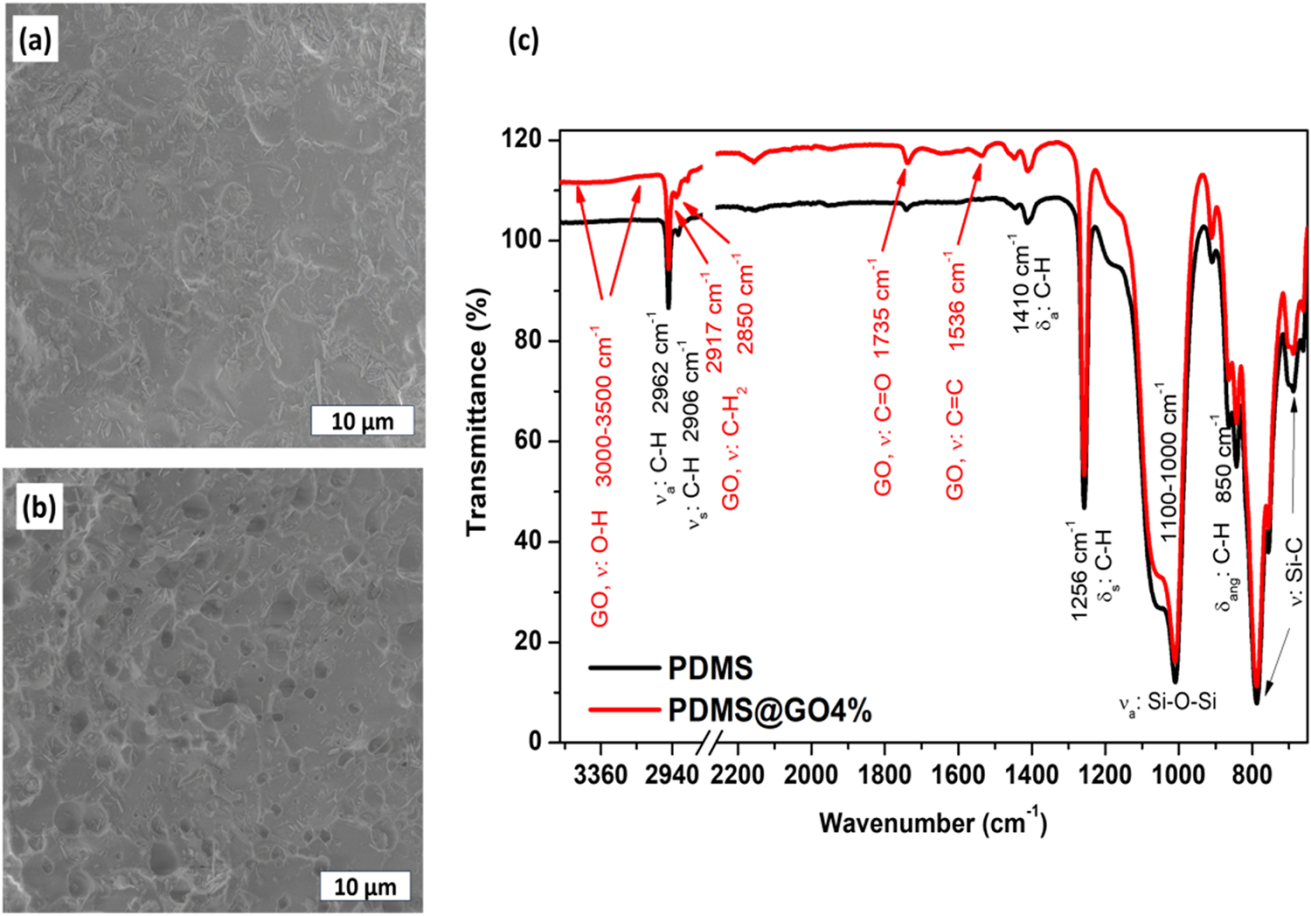
SEM images of (a) pure PDMS and (b) PDMS@GO films. (c) FTIR spectra for pure PDMS and PDMS@GO films.

In the FTIR spectrum, Figure 4c, bands at 2965 cm^–1^ and 2908 cm^–1^ were identified referred to the symmetric and asymmetric stretching of the C–H bond of the methyl groups of the polyorganosiloxane, respectively, bands at 1410 cm^–1^ and 1256 cm^–1^ attributed to asymmetric and symmetric strain modes of C–H bond, respectively, bands at 1100 cm^–1^ to 1000 cm^–1^ corresponds to asymmetric stretching of the Si–O–Si groups,^60^ at 850 cm^–1^ related to angular deformation of the C–H bond, and at 790 cm^–1^ to stretching of the Si–C bond.^60–62^ For the PDMS-4%GO nanocomposite, a slight band between 3500 cm^–1^ and 3100 cm^–1^ was observed, corresponding to the O–H vibrational mode^63^ from GO. The band at 2906 cm^–1^, in the spectrum of the pure PDMS, is shifted in the nanocomposite spectrum to 2917 cm^–1^ with concomitant appearance of the band at 2850 cm^–1^, both characteristic of CH_2_ vibrational modes of the GO.^64^ The bands at 1735 cm^–1^ and 1536 cm^–1^ correspond to the C=O and C=C stretching vibrational modes, respectively, characteristic of GO.^65^ Compared with the PDMS spectrum, the nanocomposite shows a reduction in band intensity at 1060 cm^–1^, corresponding to Si–O–Si stretching. Thus, the absence of the OH band and the change in absorption in the Si–O stretching band corroborate the interaction between the polymer/GO phases via GO-PDMS hydrogen bonds and structural intercalation of the PDMS polymer and GO layers via Si–O–C interactions.

### 3.4 TENG Performance

Figure 5 shows the output density power for the TENGs prepared using different sponge-like biochar@ZnO NRs as the positive dielectric material (E1, E2, and E3) and PDMS or PDMS@GO films as the negative dielectric material. As previously reported,^49^ adding GO to PDMS films results in higher output density power values for all TENGs due to the oxygen groups on the GO surface promoting electron transport in the PDMS film, and acting as active sites for electron pathways.

**Figure 5:**
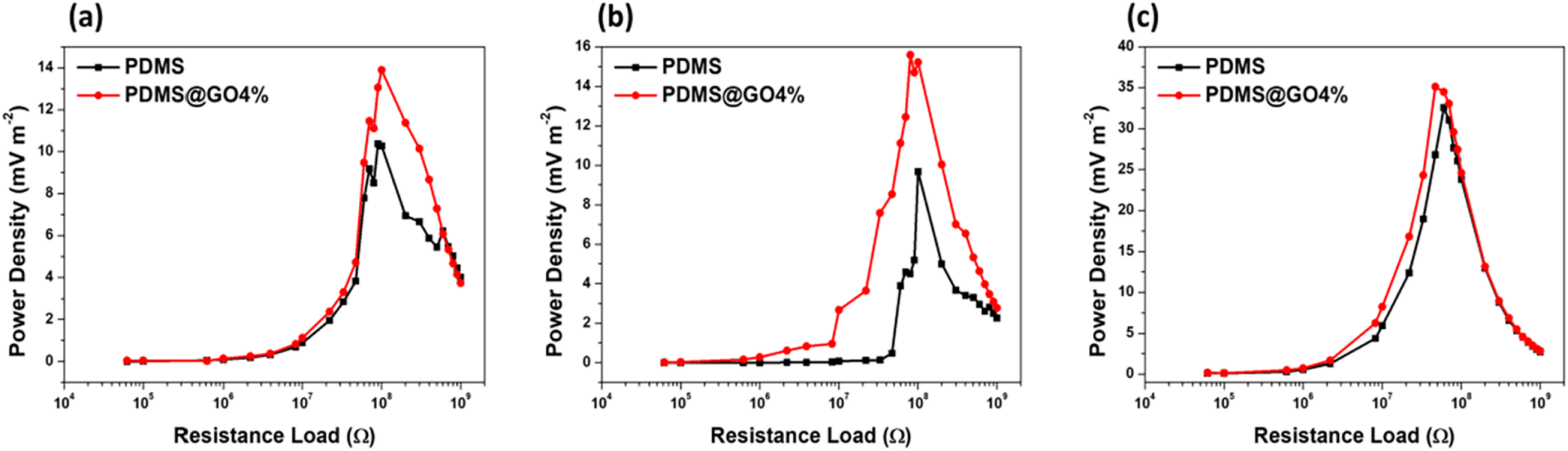
Output power density obtained from TENG devices using PDMS and PDMS@GO and different sponge-like biochar@ZnO: (a) E1, (b) E2, and (c) E3, as described in Table 1.

When we compare the use of different sponge-like biochar@ZnO NRs (E1, E2, and E3) in the TENGs, we can see an increase in the output density power from E1 to E3 related to the stability of the sponge-like structure. The E1 and E2 samples were fragile, fragmenting during handling during the assembly and electrical characterization stages. As the cycle of contact/separation continues, the material detaches from the structures, leading to poor or absent contact between the positive and negative dielectric materials. The stability of the E3 sample is attributable to the higher treatment temperature (200 °C), which is near the PVDF melting point.^66^

In the literature, it is well established that an increased β-phase content in PVDF enhances its negative charge density, making it highly effective as a negative triboelectric layer in TENG devices. Several studies have demonstrated that maximizing the β-phase improves triboelectric output by increasing surface polarization and charge trapping.^67,68^ Conversely, it has been reported that at temperatures above ∼170 °C, the β-phase content in PVDF tends to decrease, often transitioning to the nonpolar α-phase, which can reduce its triboelectric performance.^69^ In our system, FTIR analysis reveals a decline in β-phase intensity from E3 to E2 and E1, consistent with a thermally induced phase transition. Since our device architecture uses the biochar@ZnO composite as the positive dielectric layer, this reduction in β-phase (and therefore in negative charge density) helps explain the observed output trends, supporting the interpretation that PVDF’s β-phase behavior plays a secondary but coherent role in this configuration. Because the TENG built with the E3 sample as the positive dielectric material and the PDMS@GO film as the negative dielectric material exhibited a higher power density, this configuration was used for further characterization.

Figure 6a illustrates the voltage, current, and power density results obtained by the TENG in response to varying resistance loads. The optimal power density, voltage, load, and current were 35.11 mW.m^–2^, 7.6 V, 47 MΩ, and 0.16 μA, respectively. We can also evaluate the triboelectric charge density (σ) with the device output current of 0.16 μA under a 60 Hz vertical contact-separation mode. Assuming an active contact area of 7 × 5 mm^2^ (3.5 × 10^–5^ m^2^), the transferred charge per cycle (*Q* = I/f)^70^ is estimated as *Q* ≈ 2.67 × 10^–9^ C, resulting in a surface charge density (σ = *Q*/A) of σ ≈ 76.19 μC·m^–2^.^71^ This parameter is a fundamental metric for evaluating triboelectric nanogenerator (TENG) performance, directly influencing device’s ability to generate electrical output. Although the value obtained for our system is lower than those reported for high-performance conventional TENGs, which often exceed 1000 μC·m^–2^ ^71^ and can reach up to 10,000 μC·m^–2^ in devices with optimized dielectric materials and operating configurations,^72,73^ it is closer to the values reported for biochar- or biomass-derived TENGs. As summarized in Table 2, these systems typically exhibit surface charge density in the range 10–100 μC·m^–2^.

**Figure 6:**
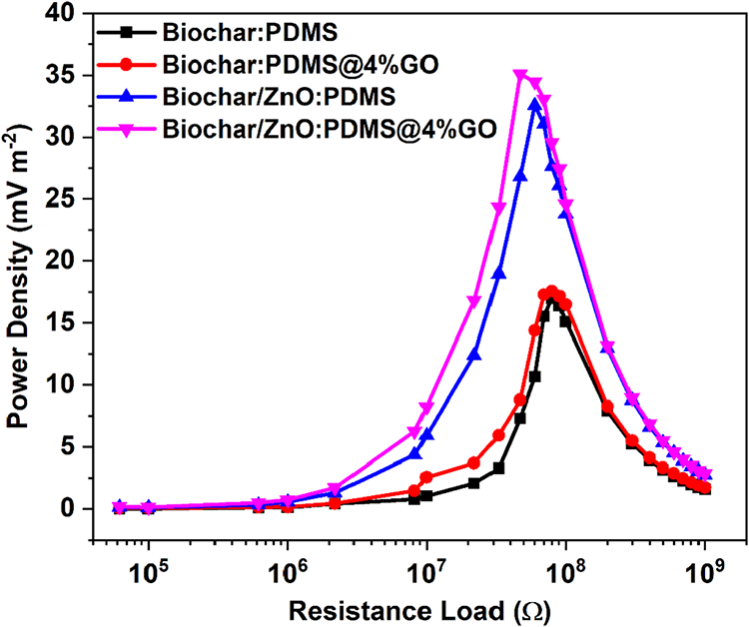
Output power density obtained from TENG devices using PDMS and PDMS@GO and different sponge-like biochar compositions: Biochar:PDMS, Biochar:PDMS@4%GO, Biochar/ZnO/PDMS, and Biochar/ZnO/PDMS@4%GO.

**Table 2:** Comparison of Materials, Structure, Voltage, Current, Power Density, and Charge Density between Various Biochar/Carbon-Based TENGs

material	structure/mode	frequency (Hz)	resistance Load (MΩ)	voltage (V)	current (μA)	charge density (μC/m^2^)	power density (mW/m^2^)	refs
Biochar@ZnO/PDMS@GO	vertical contact-separation	60	47	7.6	0.16	76.19	35.11	this work
Alc-S5-CNF/PVDF	vertical contact-separation	20	10	7.9	5.13	11.53	101.3^a^	^74^
PEO/CCP-4/PDMS	vertical contact-separation	3	2 3 60	222.1	4.3	39.7	217.3	^75^
0.5 M NaOH 6% glycerol	vertical contact-separation	0.5		3.5 V/cm2	23 nA/cm2		1.735^a^	^76^
1:9lignin-starch/Kapton								
aloe Vera (AV) film:	vertical contact-separation		10	32	0.11	102^a^	1.9^a^	^77^
PDMS								
leaves powder/PET	vertical contact-separation		20	3.86	3.78		189^a^	^78^
leaf powder/PLL/PVDF	vertical contact-separation	5	100	1000	60	100	4.47^a^	^79^
lignin/Cellulose/Citric Acid/PTFE	vertical contact-separation	1	80	335	9.74	71.45	3800	^80^
lotus-root-derived porous carbon (PC)/PDMS	vertical contact-separation	1		22.8	0.23		31	^81^

a Values estimated by the authors based on the results reported in the referenced work.

To better understand the contributions of ZnO NRs and GO to the overall performance of the device, we compared the power density outputs of different configurations, as shown in Figure 6. All devices use biochar as the base material but differ in their incorporation of ZnO and/or GO.

The device containing biochar/ZnO/PDMS@4%GO exhibits the highest peak power density, followed closely by biochar/ZnO/PDMS, indicating that ZnO enhances the energy generation. This improvement may arise from ZnO’s role as both a surface modifier, improving charge-transfer efficiency, and a piezoelectric material, potentially generating additional charge under mechanical stress.

Furthermore, both GO-containing samples show higher power densities than their GO-free counterparts. This trend supports the literature findings that GO enhances the negative triboelectricity and charge-trapping capabilities of PDMS-based composites. The GO likely increases the effective electronegativity of the negative dielectric, further boosting the triboelectric charge generation.

To ensure the reliability of our results, we conducted a repeatability test, as shown in Figure 7b. The characteristic curve response obtained for TENG devices, with the voltage data showing almost identical results in triplicate measurements, confirms the excellent stability of our as-prepared TENG device. The long-term operational stability of the fabricated TENG device was evaluated over more than 15,000 continuous mechanical contact–separation cycles. As shown in Figure 7c, the output voltage remained stable throughout the test (∼300 s), indicating robust device performance and mechanical durability. The inset of the figure presents a zoomed-in view of the open-circuit voltage (*V*_oc_) waveform, and Figure 7d shows closed-circuit current (*I*_sc_) outputs of our as-prepared TENG device. The similarity of the *V*_oc_ values indicates that the signal obtained by the circuit built for this work is saturated. The TENG produced a maximum *V*_oc_ of 12 V and an *I*_sc_ of 0.16 μA. The device was also tested for its ability to charge a 1000 μF capacitor. As shown in Figure S3, a voltage of 0.75 V was reached after 4 h of uninterrupted operation.

**Figure 7:**
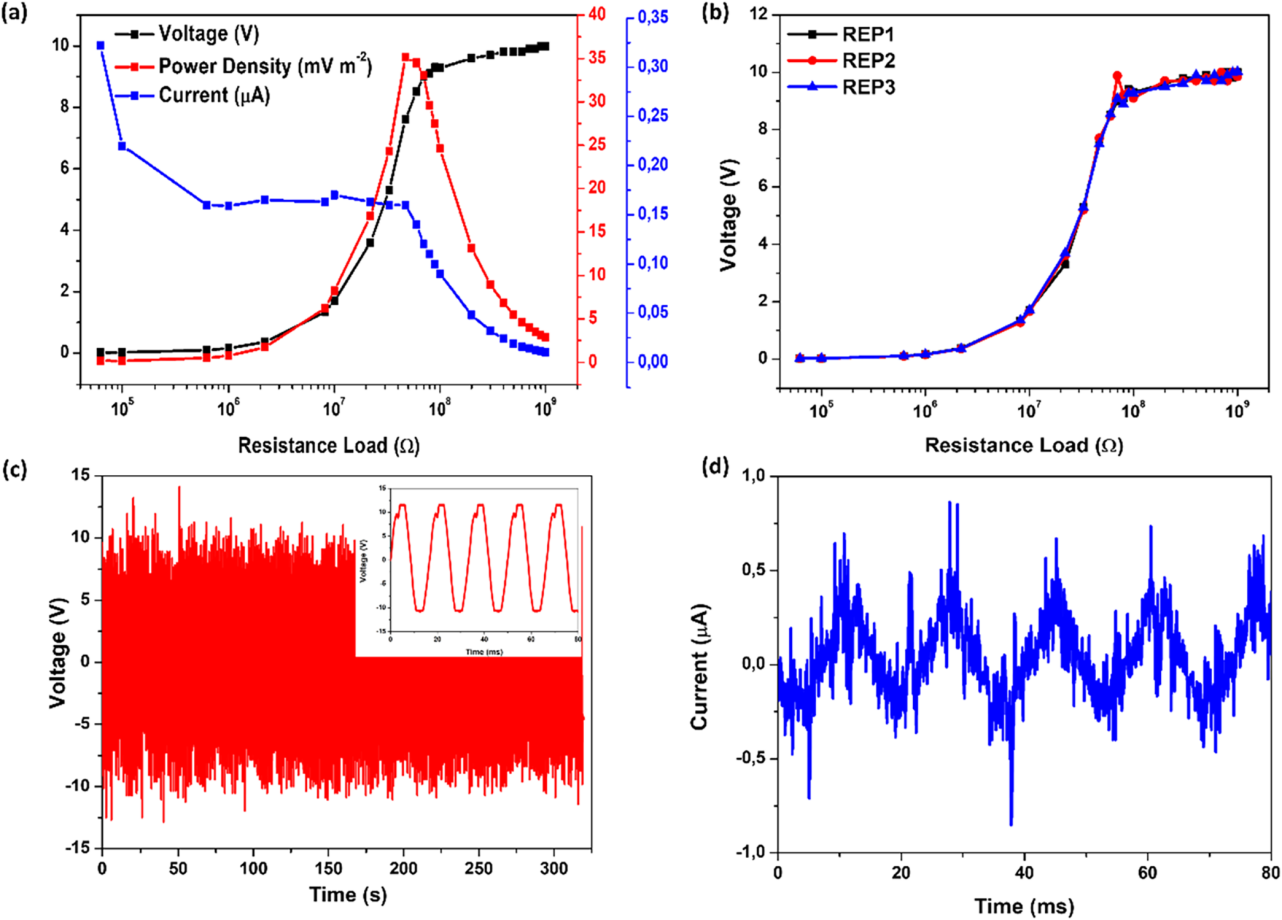
(a) Output power density, current, and voltage regarding resistance load variation. (b) Repeatability test performed in the TENG devices. (c) Stability graph showing over 15,000 cycles (inset: few cycles from the stability graph (*V*_oc_)) and (d) *I*_sc_.

Although our device’s output is lower than that of high-performance triboelectric systems based on surface-engineered ZnO or GO–PDMS composites, it is higher than that typically reported for TENGs derived from biochar or other biomass-based materials, as shown in Table 2.

Importantly, note that our system was designed not solely for maximized output but also for dual functionality, integrating energy harvesting and photocatalytic pollutant degradation. This trade-off in triboelectric output is thus balanced by the added environmental remediation capability. Moreover, the use of sustainable, low-cost materials such as sugarcane-derived biochar reinforces a balance between sustainable material use and functional performance, positioning our system above the average within the eco-friendly TENG category.

### 3.5 Photocatalytic Performance

The ZnO@biochar E3 composite (Table 1) was selected for subsequent photocatalytic evaluations owing to its superior triboelectric performance (Figure 5c), thereby ensuring optimal conditions for photocatalytic activity testing. To verify its efficacy, both adsorption and degradation experiments were conducted. Initially, an absorption test was performed in the absence of light and with the TENG off. Under these conditions, a reduction of approximately 2% in dye concentration was observed after 120 min, attributable to the biochar’s inherent adsorption capacity (Figure 8a). A photolysis test was also performed to verify the dye’s stability under light irradiation. The results showed negligible degradation (< 2%) in the absence of the TENG device, confirming that the light source contributed minimally to the overall removal efficiency.

**Figure 8:**
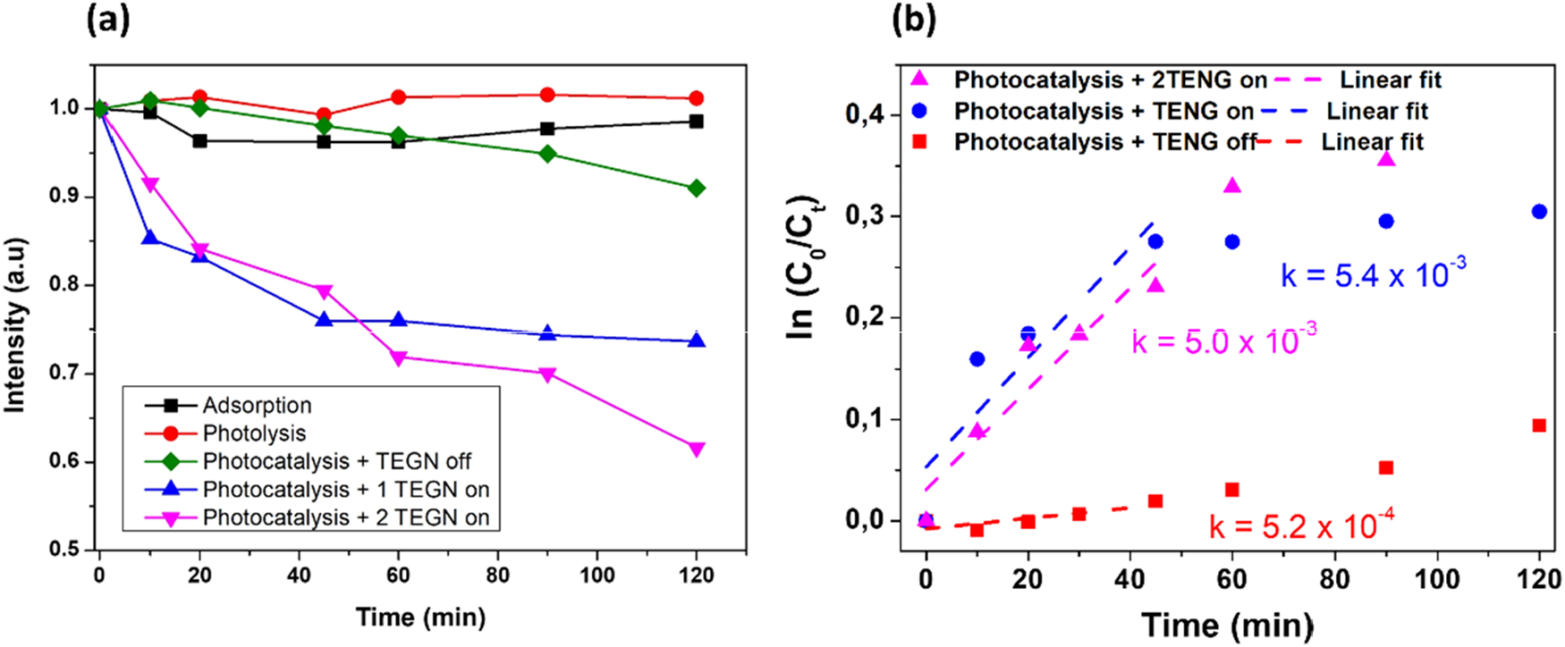
(a) Performance of the system under different conditions: adsorption, photolysis, photocatalysis + TENG Off, photocatalysis +1 TENG On, and photocatalysis +2 TENG On. (b) Linear fit of the photocatalytic data based on the pseudo-first-order kinetic model, from which the reaction rate constant (k) was determined.

The photocatalytic performance was first evaluated under light irradiation with the TENG off. Under these conditions, the system achieved a degradation efficiency of 9% for MB. When the TENG is turned on, the photodegradation efficiency increased significantly, reaching 26%. Furthermore, the introduction of an additional TENG device submerged in the MB solution increased the efficiency to approximately 42%. Although a proportional increase in photocatalytic efficiency was expected with the addition of a second TENG, the improvement was only around 15% compared to the single-TENG setup (Figure 9a). Several factors may influence photocatalytic performance in a TENG-based system, including the effectiveness of the contact-separation cycles, charge-collection efficiency, and resonance-frequency alignment. In the assembled experimental setup, slight variations, such as differences in the force applied to the steel spring or height mismatches between dielectric components, may have introduced inconsistencies. As a result, the two TENG units may not have operated synchronously at their optimal resonance frequencies, limiting the expected additive effect. Another factor that can enhance photocatalytic performance is the local turbulence generated by the TENGs’ vibration. This turbulence promotes a higher dispersion rate of the micropollutant degradation products, thereby facilitating access of the raw MB molecules to the surface of the dielectric material. Nevertheless, this study is the first to report the enhanced micropollutant degradation efficiency using a TENG system based on a sponge-like biochar@ZnO NR composite, demonstrating its potential for photocatalytic applications.

**Figure 9:**
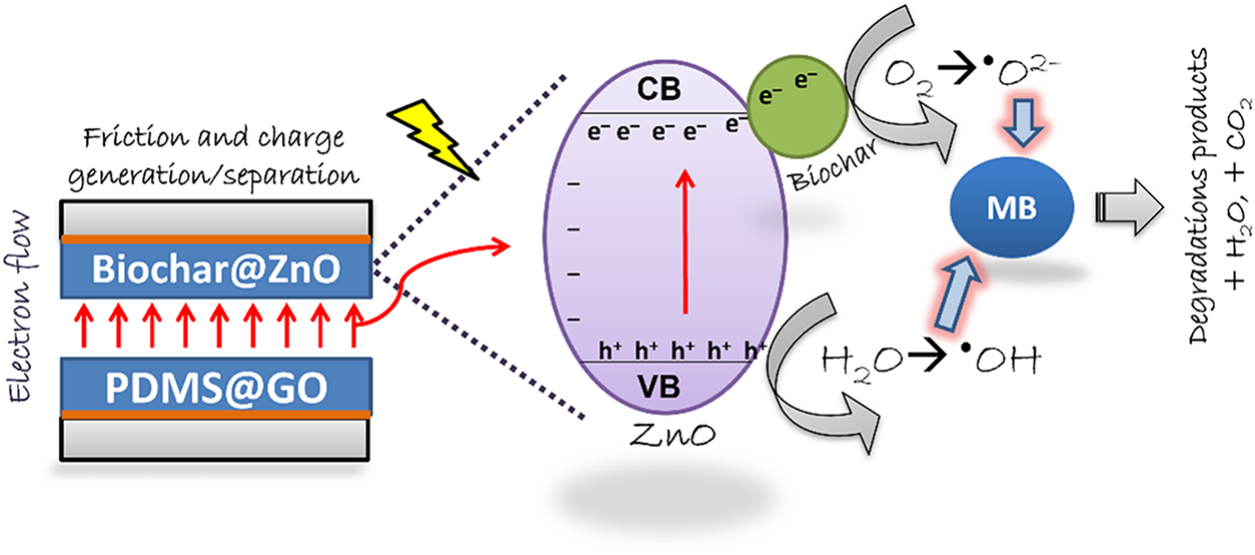
Schematic representation of electron flow and mechanism for organic pollutant photodegradation.

Figure 9b presents the fit of the data points for the photocatalysis results. In the heterogeneous photocatalytic process involving the TENG system, the photocatalysis follows a pseudo-first-order kinetic model reaction. This behavior is consistent with the Langmuir–Hinshelwood mechanism, as described by eq 3, and is attributed to the high water content and the low micropollutant concentration. The linear correlation observed in Figure 9b supports the pseudo-first-order kinetics, and the rate constant (*k*) can be determined from the slope of the fitted straight line.

3 $$\ln\frac{C_{0}}{C_{t}}=\mathrm{kt}$$

where *C*_0_ and *C*_*t*_ are the initial and at a given time concentrations, and *k* is the rate constant.

From Figure 8b, it is observed that the reaction rate constant (*k*) increases by a factor of 10 when photocatalysis is performed with the TENG on. However, it is evident that adding a second TENG does not further enhance the reaction kinetics. Instead, its main contribution lies in the system’s ability to degrade a greater amount of dye as the photocatalysis time extends beyond 60 min (Figure 8).

Table 3 compares our results to those of other TENG-based devices used for contaminant degradation. Analysis of the experimental protocols indicates that other studies often employ higher-power light sources or add electrolytes to the solution to enhance degradation kinetics. Notably, even under lower-intensity irradiation in our research, our system achieved a 42% degradation rate in 2 h, outperforming other works that reported significantly lower efficiencies after 5 h of operation. This enhanced efficiency, driven by the synergistic effect of the TENG’s triboelectric potential in separating photogenerated charges, underscores the potential of TENG-driven photocatalysis as a promising technology and sustainable approach for environmental self-cleaning applications, including the degradation of persistent and harmful pollutants.

**Table 3:** Comparison Results of Degradation Efficiency from Some TENG Devices

TENG configuration	synthesis conditions	experimental procedure	efficiency	refs
Biochar@ZnO/PDMS@GO	- Biochar calcined at 750 °C/4 h;	- methylene blue (4 mg/L);	42% in 2 h	this work
	- ZnO_2_ NRs grown onto biochar surface through the chemical bath deposition method at 90 °C/2 h;	- LED flexible ring light, 30 W;		
	- PDMS mixed with graphene Oxide and deposited by spin coating and cured at 95 °C.	- mechanical external force at 60 Hz;		
Cu_2_O/Bi_2_MoO_6_/Pt	- Cu_2_O electrochemically deposited onto an ITO substrate and annealed at 300 °C;	- Tetracycline hydrochloride (10 mg/L);	49% in 2 h	^82^
	- hydrothermal synthesis of Bi_2_MoO_6_ at 80 °C/10 min;	- rotor speed 100 r/min;		
		- 300 W xenon lamp		
TiO_2_-PTFE-Al/Pt	- commercial TiO_2_;	- methyl orange (20 mg/L);	76% in 2 h	^37^
		- irradiation with a solar simulator (500 W)		
Biochar@G/Pt	- Biochar and graphene (8:1) mixed by ball milling;	- methyl Orange (6 ppm);	90% in 5 h	^83^
	- addition of 0.5 mL of 60 wt % PTFE adhesive;	- electro-Fenton system: Fe^2+^ (2 mM), Na_2_SO_4_ (0.05 M) as the electrolyte		
	- dropped onto a stainless-steel mesh and put into an oven to dry at 105 °C/12 h.			
carbon/PTFE	- Biochar calcined at 600 °C/2 h;	- methyl Red (10 mg/L);	96% in 4 h	^84^
	- chemical activation with KHCO_3_ at 800 °C/2 h;	- HCl electrolyte (0.5 mol/L)		
Cu_2_WS_4_/PTFE	- hydrothermal synthesis of Cu_2_WS_4_ at 200 °C/72 h;	- mixed solution of NaCl and Rhodamine B;	∼22% in 3 h;	^85^
	- mixed with DMF and PVDF, followed by deposition on Ni foam by spin coating and dried at 70 °C/1 h;	- addition of isopropyl alcohol (IPA) and hydrogen peroxide (H_2_O_2_) with a dosage of 0.2 mmol/L.	∼50% in 10 h;	
			∼100% in 20 h	
LaFe-ZnO_2_/PDMS	- hydrothermal synthesis of La,Fe codoped ZnO_2_ at 180 °C/12 h;	- mixed solution of NaCl and Rhodamine B;	∼30% in 5 h	^86^
	- PDMS deposited on Cu films by spin coating and dried at 95 °C.			
BaTiO_3_-ZnO_2_/PET-ITO	- BTO synthesized via the thermostatic water-bath method at 90 °C/4 h under continuous stirring and static aging for 24 h;	-methyl Orange (30 mg/L);	∼20% in 3 h;	^87^
	- BTO added into ninc nitrate aqueous solution for hydrothermal synthesis at 105 °C/16 h;	- addition of NaCl;	∼30% in 5 h	
	- drying at 80 °C for 6 h;	- Cu electrodes		
	- BTO-ZnO mixed with PDMS, followed by deposition on ITO by spin coating and cured at 90 °C for 20 min			

The degradation mechanism of a micropollutant through heterogeneous photocatalysis relies on the absorption of electromagnetic radiation by the semiconductor material. This absorption, equivalent to or higher than the bandgap energy, triggers the electron excitation from the valence band to the conduction band, consequently generating the charge pair of electron–hole (e^–^/h^+^). The efficiency of photocatalytic activity relies on the efficient and long-lasting separation of e^–^/h^+^ pairs, which promotes reactions with aqueous medium species to reduce species as O_2_ to superoxide radicals, O_2_^•–^, and/or oxidize H_2_O or hydroxyl, OH^–^ to hydroxyl radicals OH^•^. These radicals degrade pollutant molecules. Moreover, employing visible-light-activated photocatalysts reduces the costs associated with system implementation. In this study, a notable enhancement in the photocatalytic efficiency for dye degradation was observed when the TENG was used in conjunction with photocatalysis. This improvement can be attributed to the strong electric field generated during TENG’s operating mode, facilitating the separation of charge carriers (e^–^/h^+^) to the photocatalyst’s surface,^88,89^ increasing its efficiency in the degradation of the micropollutant, even using light in the visible region for its activation.

In Figure 9, we present a diagram illustrating electron flow from charge generation and separation (triboelectric effect due to friction between the layers) and from photogenerated electrons (due to lamp irradiation). In the biochar@ZnO composite, the photoexcited electrons from the valence band to the conduction band of the semiconductor (ZnO) are captured by biochar and react with adsorbed oxygen to form the reactive species such as the superoxide radical, ^•^O^2–^. Thereafter, the superoxide radical can be transferred to organic pollutants, enabling efficient degradation. The composite promotes the separation of photogenerated electron–hole pairs. The biochar composite acts as the positive dielectric material, and the PDMS@GO film as the negative dielectric material. The charge separation due to the e^–^/h^+^ pair is one of the critical aspects of photocatalysis, leading to the production of reactive oxygen species such as ^•^OH and ^•^O^2–^ in the aqueous environment, potentially leading to complete mineralization.

## 4 Conclusions

This study demonstrated the fabrication of a sponge-like triboelectric nanogenerator (TENG) using a sponge-like biochar@ZnO NR array as the positive dielectric material and a PDMS@GO composite as the negative dielectric material. The device exhibited a power density of 35.11 mW/m^2^, an output voltage of 7.6 V, a load resistance of 47 MΩ, and a current of 0.16 μA. The TENGs also showed excellent reproducibility, delivering nearly identical voltage outputs across triplicate measurements, and were able to charge a 1000 μF capacitor within 4 h. Furthermore, the TENG was successfully integrated into a photocatalytic system, leading to a significant improvement in the methylene blue degradation efficiency. When two TENG units were employed, the degradation efficiency reached approximately 42%, highlighting the synergistic interaction between the TENG’s energy harvesting mechanism and the photocatalytic process. These findings demonstrate the potential of this hybrid system for sustainable energy conversion and environmental remediation. Future studies should focus on optimizing material composition, refining device architecture, and improving system integration to further enhance efficiency and ensure long-term operational stability. Advancements in these areas could play a pivotal role in the development of next-generation technologies for energy harvesting and environmental cleanup.

## Supplementary Material



## Notes

FTIR characterization of the raw biomass and biomass after acid treatment; SEM micrographs at various magnifications; a graph illustrating the charging of a 1000 μF capacitor obtained using the TENG device; and photographs of the experimental setup employed for MB degradation (PDF)
