# Supplementary material for: Multifunctional Sponge-like Biochar@ZnO Nanorods Material: Applications in Triboelectric Nanogenerators to Enhance Photocatalysis
Source: ACS Omega. 2026 Feb 16;11(8):13445–58. doi: 10.1021/acsomega.5c10833 (PMC12961561; doi:10.1021/acsomega.5c10833)
Supplement: Supplementary file 1 [file ao5c10833_si_001.pdf]

Multifunctional sponge-like biochar@ZnO nanorods material: applications in triboelectric nanogenerators to enhance photocatalysis

Agnes Nascimento Simões[1,2], Rafael Aparecido Ciola Amoresi[3], Glauco Meireles Mascarenhas Morandi Lustosa[2], Waldir Antonio Bizzo[1], Talita Mazon[2*]

[1]*Faculdade de Engenharia Mecânica, Universidade Estadual de Campinas, Departamento de Energia (DE), R. Mendeleyev, 200 - Cidade Universitária, Campinas - SP, 13083-860, Campinas – São Paulo, Brazil.*

[2]*Centro de Tecnologia da Informação Renato Archer, Ministério da Ciência, Tecnologia e Inovação (MCTI), CTI, Rod. D. Pedro I, KM 143.6, 13069-901 Campinas, São Paulo, Brazil*

[3]*Departamento de Química Física y Analítica, Universitat Jaume I, Castellón de la Plana, Av. Sos Baynat, s/n, 12071, Spain*

**talita.mazon@cti.gov.br*

Support Information

Figure S1 shows the FTIR spectra of raw sugarcane bagasse biomass and the sample after acid treatment. This step aims to reduce the degree of polymerization and create a porous surface. Some bands overlap between the two samples, for example, at 1730 cm^{-1}, 1610 cm^{-1}, 1515 cm^{-1}, 1020 cm^{-1}, and 825 cm^{-1}. These bands can be attributed to the C=O stretch of hemicellulose structure, the aromatic stretch of lignin molecule, the C=C bond of aromatic compounds also in lignin molecule, the C-O-C vibration characteristic of cellulose and hemicellulose molecules, and aromatic ring stretch of the lignin, respectively (Chandel et al. 2014; Portero-Barahona et al. 2019; Quiroga et al. 2020; Thite e Nerurkar 2019; Brant et al. 2020). It is also noted that, referring to these bands, there is an intensity reduction of these bands on the acid-treated biomass sample. Besides, the other two important bands are observed around 3300 cm-1 and 2900 cm-1. The first can be attributed to the -OH stretch of the water molecule and the hydroxyl groups characteristic of cellulose and lignin are highly reduced. Also, the second band corresponds to the stretch of $-CH_2$ and $-CH_3$ radicals present in the main components of pure biomass; the almost disappearance of this band could indicate that these bonds were broken during the acid treatment (Thite e Nerurkar 2019; Brant et al. 2020; Quiroga et al. 2020; Portero-Barahona et al. 2019; Chandel et al. 2014).

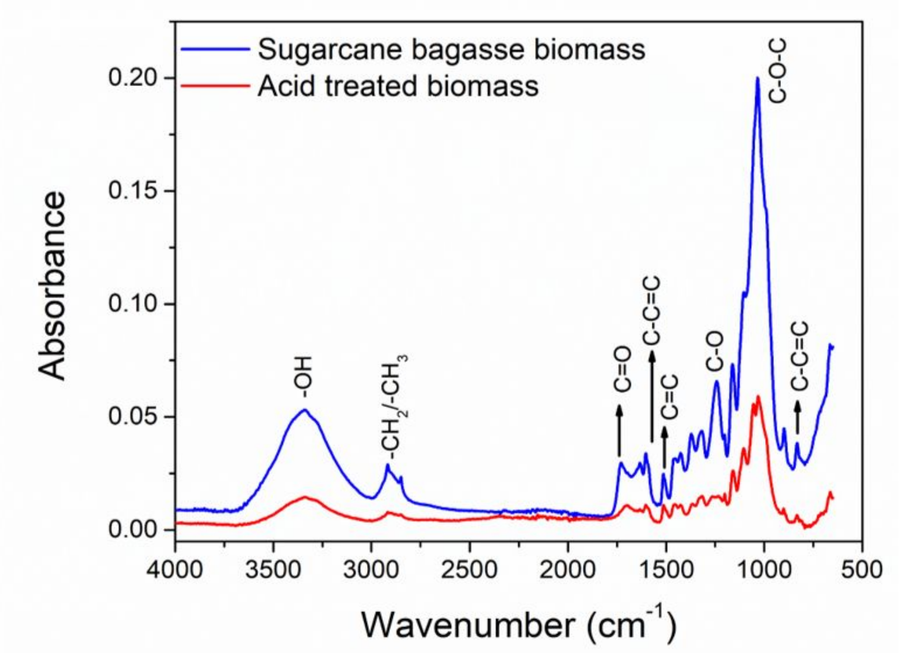

Figure S1. FTIR analyses of sugarcane bagasse biomass before and after acid treatment.

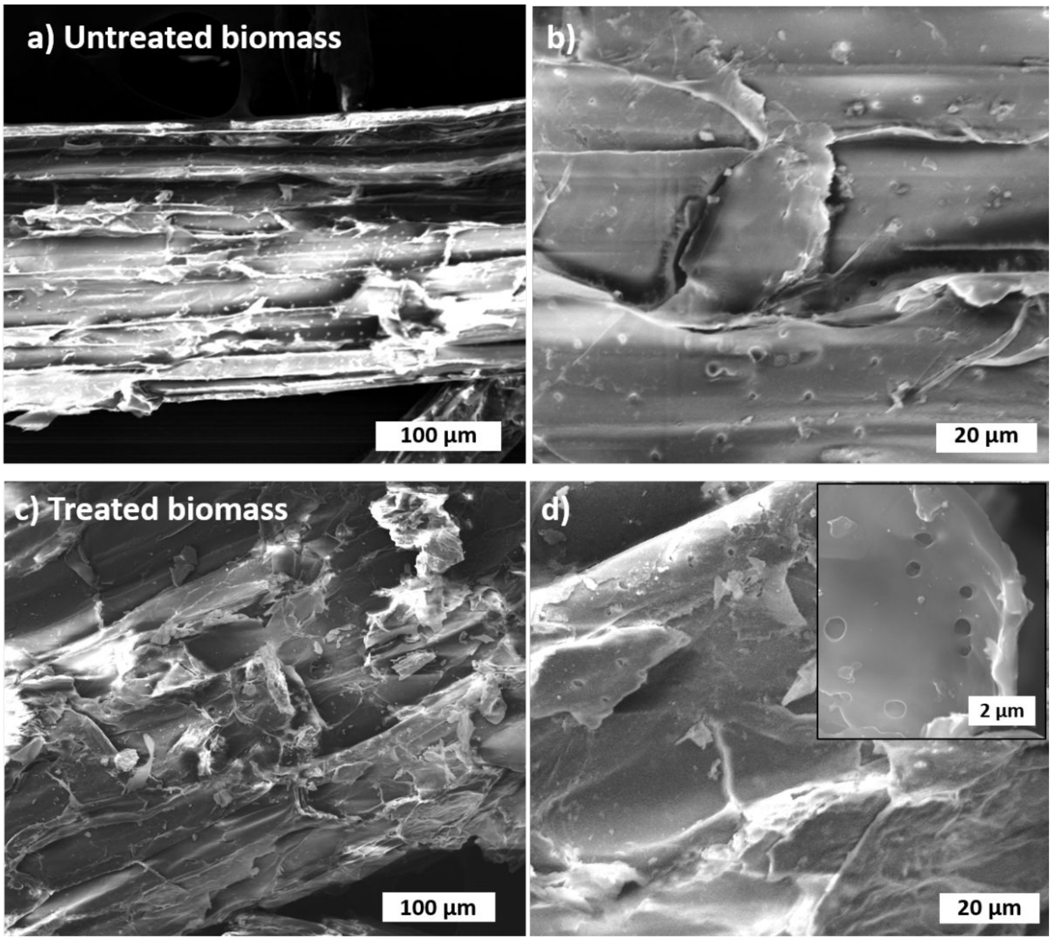

Figure S2. SEM images of sugarcane bagasse biomass (a,b) before and (c,d) after acid treatment.

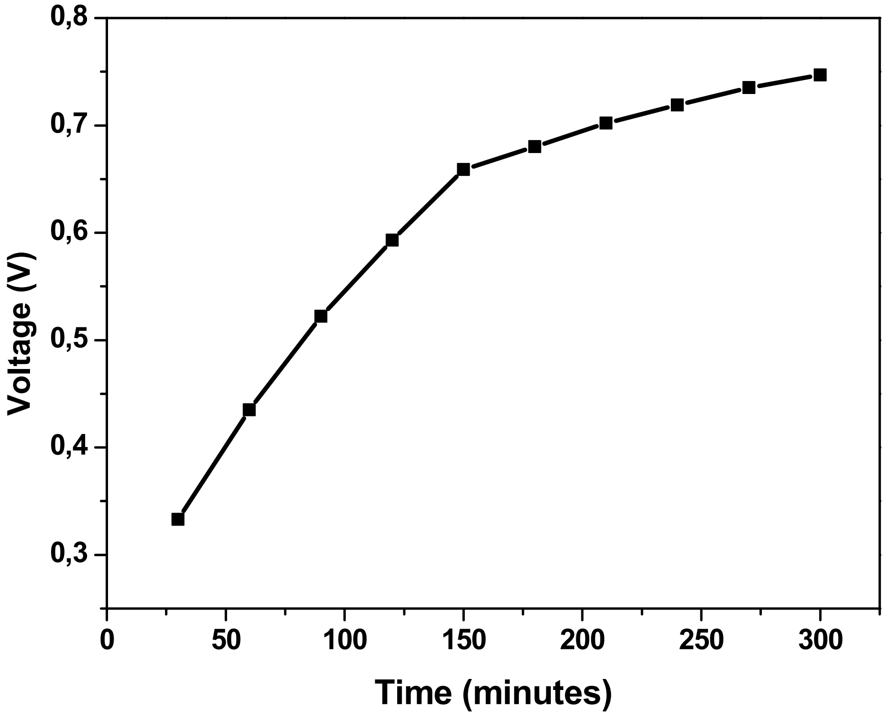

Figure S3. Charging capacitor test result obtained from TENG device using the sponge-like biochar:ZnO NRs composite as the positive dielectric material and PDMS:GO 4% film as the negative dielectric material.

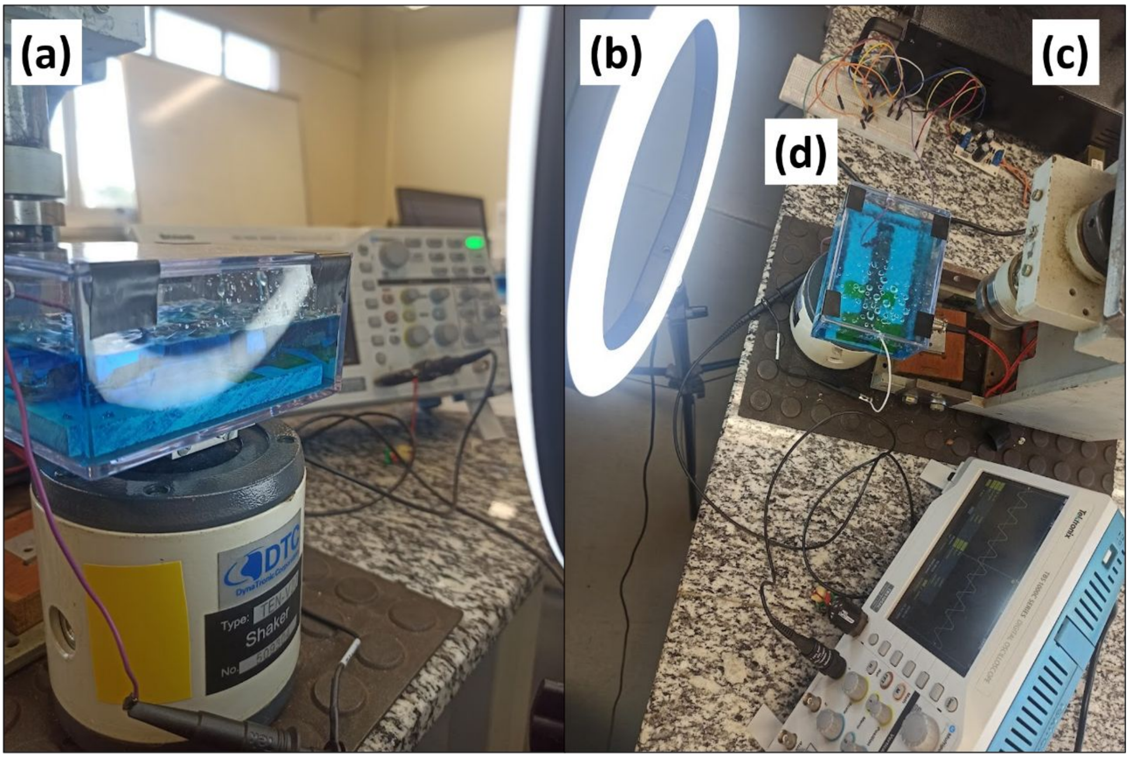

Figure S4: (a) Front view of the setup: shaker responsible for the mechanical vibration input; transparent acrylic recipient containing polymeric base where the TENG is affixed fill with the methylene blue solution; in the background the oscilloscope responsible for electrical measurements; (b) top view of the setup: on the right, the ring light responsible for the light input; (c) amplifier responsible for the frequency and gain input; (d) electrical circuit responsible for converting the TENG signal output into electrical signal for the oscilloscope.

References

Brant, Antônio Jedson Caldeira, Natália Naime, Ademar Benévolo Lugão, e Patrícia Ponce. 2020. “Cellulose Nanoparticles Extracted from Sugarcane Bagasse and Their Use in Biodegradable Recipients for Improving Physical Properties and Water Barrier of the Latter”. *Materials Sciences and Applications* 11 (01): 01. https://doi.org/10.4236/msa.2020.111007.

Chandel, Anuj K., Felipe AF Antunes, Virgilio Anjos, et al. 2014. “Multi-scale structural and chemical analysis of sugarcane bagasse in the process of sequential acid–base pretreatment and ethanol production by Scheffersomyces shehatae and Saccharomyces cerevisiae”. *Biotechnology for Biofuels* 7 (1): 63. https://doi.org/10.1186/1754-6834-7-63.

Portero-Barahona, Patricia, Enrique Javier Carvajal-Barriga, Jesús Martín-Gil, e Pablo Martín-Ramos. 2019. “Sugarcane Bagasse Hydrolysis Enhancement by Microwave-Assisted Sulfolane Pretreatment”. *Energies* 12 (9): 9. https://doi.org/10.3390/en12091703.

Quiroga, Eliana, Julia Moltó, Juan A. Conesa, Manuel F. Valero, e Martha Cobo. 2020. “Kinetics of the Catalytic Thermal Degradation of Sugarcane Residual Biomass Over Rh-Pt/CeO2-SiO2 for Syngas Production”. *Catalysts* 10 (5): 5. https://doi.org/10.3390/catal10050508.

Thite, Vihang S., e Anuradha S. Nerurkar. 2019. “Valorization of Sugarcane Bagasse by Chemical Pretreatment and Enzyme Mediated Deconstruction”. *Scientific Reports* 9 (1): 15904. https://doi.org/10.1038/s41598-019-52347-7.
